# Efficacy of Acute Intermittent Hypoxia on Physical Function and Health Status in Humans with Spinal Cord Injury: A Brief Review

**DOI:** 10.1155/2015/409625

**Published:** 2015-06-08

**Authors:** Todd A. Astorino, Eric T. Harness, Ailish C. White

**Affiliations:** ^1^Department of Kinesiology, CSU San Marcos, San Marcos, CA 92096-0001, USA; ^2^Neuro Ex, Oceanside, CA, USA

## Abstract

Spinal cord injury (SCI) results in a loss of motor and sensory function and is consequent with reductions in locomotion, leading to a relatively sedentary lifestyle which predisposes individuals to premature morbidity and mortality. Many exercise modalities have been employed to improve physical function and health status in SCI, yet they are typically expensive, require many trained clinicians to implement, and are thus relegated to specialized rehabilitation centers. These characteristics of traditional exercise-based rehabilitation in SCI make their application relatively impractical considering the time-intensive nature of these regimens and patients' poor access to exercise. A promising approach to improve physical function in persons with SCI is exposure to acute intermittent hypoxia (IH) in the form of a small amount of sessions of brief, repeated exposures to low oxygen gas mixtures interspersed with normoxic breathing. This review summarizes the clinical application of IH in humans with SCI, describes recommended dosing and potential side effects of IH, and reviews existing data concerning the efficacy of relatively brief exposures of IH to modify health and physical function. Potential mechanisms explaining the effects of IH are also discussed. Collectively, IH appears to be a safe, time-efficient, and robust approach to enhance physical function in chronic, incomplete SCI.

## 1. Introduction

Incidence of spinal cord injury (SCI) is equal to up to 83 cases per million persons worldwide [1] and in the United States, 12,000 new cases occur each year [1]. Approximately 33 percent of spinal cord injuries are complete [1] with minimal motor or sensory function below the level of injury, and approximately 60% of the injuries are classified as tetraplegia [1]. Consequent with SCI onset are severe financial, physical, and psychological effects. For example, expected health care costs in the first year after injury range from $334,000 to over $1,000,000 [2], and employment is low (only 11.5% employed at year 1) leading to loss of income [1]. Due to autonomic impairments as well as deficits in mobility and sensation, onset of SCI leads to inactivity which diminishes health status. Muscle atrophy [3] and bone loss [4] are marked early after SCI and are especially dramatic in the trunk and lower extremities located below the level of injury. These deleterious changes increase risk of fractures and overuse injuries [5]. Furthermore, there is a shift to more fast twitch muscle fiber expression which reduces muscle oxidative capacity and accelerates fatigability [6]. Chronic pain [7] and depression [8] are also concurrent with SCI which tend to reduce quality of life in this population. Recent data show that two of the leading causes of morbidity and mortality in persons with chronic SCI are cardiovascular disease and type 2 diabetes [9] which is partially attributed to their increasing life span which is approaching that of able-bodied individuals. Therefore, practical and effective exercise regimens are needed to modify physical function and improve health status in this population.

Many exercise modalities have been shown to significantly improve physical function and modify chronic disease risk in SCI, including arm ergometry [10], functional electrical stimulation exercise (FES) [11], resistance training [12], locomotor training [13], and more recently vibration training [14] and activity-based therapy [15, 16]. Nevertheless, with exception of arm ergometry and FES, these modalities are typically expensive and localized to a few specialized facilities. They also require many trained personnel to operate equipment and/or monitor training, and many months of training tend to elicit relatively small gains in variables including bone mineral density [16] and body composition [17] which are dramatically altered after SCI and thus commonly identified as targets of rehabilitation to improve health status. So despite the documented success of exercise-based rehabilitation in promoting health-based outcomes, it may be somewhat impractical for most persons with long-term SCI to access. Moreover, it is reported that participation in regular physical activity in persons with chronic SCI is low [18], leading to low values for cardiorespiratory fitness expressed as maximal oxygen uptake [19] which is detrimental to their health and overall function. Consequently, it is evident that other approaches to improve health status in SCI should be identified. Remote ischemic preconditioning (RIPC) has been shown to protect the spinal cord against ischaemic injury [20], yet its application is widely confined to animal-based models of SCI. Moreover, examination of efficacy of RIPC in the human spinal cord to elicit neuroprotection seems to be in its infancy, although some benefits have been identified [21].

Despite the challenges faced by persons with SCI to follow an active lifestyle, neural recovery after injury does occur, which is referred to as neuroplasticity. Neuroplasticity refers to the ability of the central nervous system to respond to damage or new stimuli through regeneration, reinnervation, and/or remodeling of neural circuitry. After SCI, neuroplasticity plays an important role in adapting to and recovering function below the level of the injury. One of the possible mechanisms is through exercise or activity-induced changes in neurotrophin expression. It has been shown that neurotrophins, particularly brain-derived neurotrophic factor (BDNF), have at least a transient increase in expression during and after acute exercise [22]. Similar increases in BDNF were also shown in trained men with SCI during exercise [23]. Brain-derived neurotrophic factor is a protein of 252 amino acids and is coded by the BDNF gene [24]. Research has shown that BDNF has a large range of functions including neuronal survival and protection, axonal and dendritic remodeling/growth, and neuronal differentiation and synaptic plasticity. Consequently, BDNF is a known agonist for neural protection and plasticity and may be a significant contributor to increases in neural function in response to chronic physical activity [25, 26].

## 2. Intermittent Hypoxia

One recently introduced strategy that may induce spinal plasticity is intermittent hypoxia (IH) characterized by repeated exposures to low oxygen levels which can be administered during a single session (acute exposure) or over more prolonged periods of time (chronic exposure). Long-term exposure to hypoxia (4–8 wk) including sojourn to altitudes ranging within 4,000–5,000 m has been reported as being detrimental in the form of reductions in exercise capacity and body weight [27], cross-sectional area of the vastus lateralis, size of type I and type II muscle fibers, activity of metabolic enzymes, and mitochondrial density [28, 29]. Nevertheless, there are early reports of use of intermittent hypoxia in the former Soviet Union [30] to treat clinical disorders ranging from psychiatric depression to hypertension. For a comprehensive summary of the neurobiology of intermittent hypoxia, the authors recommend two recent review articles [31, 32]. In addition, IH was used as a strategy to promote weight loss and enhance health status, as results from two long-term (4–8 wk) clinical trials demonstrated greater improvements in weight loss [33] and various health markers [34] in individuals performing moderate endurance training in hypoxia (15% O_2_) versus normoxia. Consequently, chronic exposure to hypoxia for a few months may help modify health status in exercising humans by maximizing various training-induced adaptations.

Animal models of SCI also suggest benefits of IH exposure. In rats with hemisection at C2, 7 days of daily 50 min exposure to AIH equivalent to 10.5% O_2_ led to significant improvements in respiratory and motor function which were consequent with improved BDNF immunoreactivity [35]. Baker-Herman et al. [36] demonstrated that intermittent hypoxia (three 5 min episodes of 11% O_2_) caused serotonin-dependent BDNF synthesis in the spine, leading to spinal plasticity in rats. Similarly, 5 days of IH (11% O_2_) in rats undergoing C2 hemisection enhanced the density of mammalian/mechanistic target of rapamycin (mTOR) [37], a protein kinase that regulates cell growth, proliferation, motility, and survival as well as protein synthesis and transcription. In addition, this brief exposure to IH reduced levels of dual phosphatase and tensin homolog (PTEN), a protein that is linked to cell growth inhibition within phrenic motor neurons [38]. It is evident that PTEN inhibits phosphoinositide-3-kinase (PI3K)/protein kinase B/mTOR signaling pathways leading to suppression of the mTOR pathway and attenuation of central nervous system axon regeneration [39, 40]. Thus, decreased levels of PTEN and increased mTOR following intermittent hypoxia lead to increased protein synthesis and enhanced plasticity and regeneration of phrenic motor neurons after SCI [41]. These results suggest that IH can be used as a time-efficient strategy to treat respiratory dysfunction following SCI.

## 3. Recommended Dose of IH and Potential Side Effects

**Figure 1:**
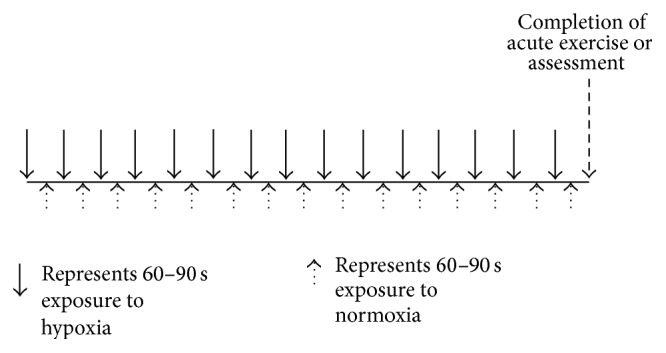
Schematic depicting the typical application of a single exposure to acute intermittent hypoxia preceding completion of exercise or an assessment in humans with SCI. Blood pressure, heart rate, and oxygen-hemoglobin saturation are continually monitored during this application to ensure normal responses to hypoxia.

Typical exposures to AIH are characterized by brief, repeated administrations of hypoxia interspersed with breathing normoxic air (Figure 1). During IH exposure, blood pressure, heart rate, and oxygen-hemoglobin saturation (S_a_O_2_) are continuously monitored to assess participants' responses to breathing low oxygen mixtures.

In a recent case study in a woman with SCI [42], eight 2 min exposures to IH equivalent to 8% O_2_ interspersed with 2 min normoxic exposures were administered daily over a period of 10 days. In a study by Trumbower et al. [43], fifteen 1 min exposures to IH equal to 9% O_2_ separated by 1 min of room air breathing were performed during a single session. A similar protocol was used in a more recent study in individuals with incomplete SCI [44], although fifteen 1.5 min exposures to 9% O_2_ separated by 1 min of normoxic breathing were employed each day over a period of 5 days. Overall, IH can be administered in a single session lasting 30–35 min using hypoxic mixtures ranging within 8-9% O_2_. In an animal model of SCI, six 5 min blocks of 11% O_2_ separated by 5 min blocks of normoxia were performed daily for five consecutive days [37]. In a recent review [45], it was suggested that modest levels of hypoxia (9–16% O_2_) and a relatively low number of exposures (3–15 episodes per day) seem to elicit beneficial effects without pathology, whereas severe hypoxia (2–8% O_2_) characterized by an extensive amount of episodes (48–2,400 exposures per day) elicits progressively greater pathology.

Despite documented deleterious effects seen with long-term exposure to hypoxia, no side effects of relatively brief (≤15 exposures per day for ≤10 d) IH exposure including spasticity, elevated heart rate/blood pressure, or autonomic dysreflexia have been reported in human-based SCI studies. In fact, this low-dose IH protocol as administered in human-based studies does not elicit hypertension, weight loss, or hippocampal gliosis or cell death in rats [44]. Nevertheless, transient decreases in blood pressure and/or oxyhemoglobin saturation are typically reported during exposure to IH (S_a_O_2_ = 81 ± 1%, [44]), yet the magnitude of these declines seems to be lower than that causing serious health issues. Consequently, the specific O_2_ fractions, minimal amount of bouts, and relatively brief duration of exposure to hypoxia used in previous studies seem safe and well-tolerated by humans with incomplete SCI, although the optimal dose of IH to maximize physical gains and to be potentially employed in complete SCI is unknown.

## 4. Effects of Short-Term IH on Physical Function in Humans with SCI

Table 1 summarizes existing research concerning effects of short-term IH on various health-related parameters in humans with SCI. One common target of IH in humans with SCI is respiratory function, as it is compromised in SCI due to disrupted neural efferent output to the respiratory muscles [46]. Data from a case study [42] in a woman with incomplete SCI demonstrated efficacy of short-term and more chronic (10 days) IH administration on respiratory function, which has been identified as a leading cause of mortality in this population [47]. Tests of pulmonary function, ventilatory load compensation, and respiratory perceptual sensitivity were conducted at baseline, after sham, and at 1 and 10 days of IH. Forced vital capacity (FVC) and forced expiratory volume in 1 second (FEV-1) were higher after sham and at days 1 and 10 of IH versus baseline, although IH did not elicit significantly greater increases than sham exposure. After 10 days of IH, ventilatory load compensation was improved versus baseline.

Respiratory function was also examined in men and women with chronic, incomplete SCI [48]. IH consisted of 10 days of eight episodes of breathing 8% O_2_, with 2 min exposures interspersed by 2 min breathing of normoxic air. Results showed that only 1 day of IH significantly increased tidal volume and respiratory rate leading to enhanced ventilation which were not seen in the sham treatment. Ten days of IH also improved FVC and FEV-1 in 50% of participants, but ventilation was not further improved. Overall, these studies show that short-term administration of IH enhances respiratory function in humans with SCI and supports data from animal studies [49]. For example, 7 consecutive days of IH exposure (ten 5 min episodes at 10.5% O_2_ interspersed with normoxia) induced motor plasticity in respiratory and nonrespiratory function without accompanying pathology in a rodent model of chronic SCI, which was associated with increases in BDNF [35].

Changes in voluntary motor function as represented by measurements of muscle torque and walking ability have also been investigated in response to short-term IH in humans with SCI. An initial study performed in humans with SCI examined the effects of a single dose of IH on ankle torque in participants with chronic, incomplete injury [43]. Exposure to IH or normoxia was separated by 2 wk as this was a randomized crossover design in which participants completed both an IH trial and control (sham). Compared to a sham trial in which normoxic air was inspired, fifteen 1 min exposures to IH led to significant improvements in maximal plantar flexion torque (82 ± 33%) and peak gastrocnemius electromyographic activity (43 ± 17%) which were observed immediately after exposure. Thirty and 60 min after exposure to IH, values were still elevated compared to those in the sham trial. In a subset of participants, maximal torque remained elevated 4 h after IH administration, suggesting long-lasting effects of this brief exposure to hypoxia. Although no mechanism was identified to explain these results, authors speculated that this may be driven by hypoxia-induced release of serotonin which initiates release of BDNF.

In a recent study examining effects of short-term IH in persons with SCI [44], 19 men and women with chronic (9 ± 2 yr) incomplete injury who were able to take at least one step without assistance were recruited. In the initial phase of the randomized crossover study, nine individuals underwent 37.5 min of IH (9% O_2_) or sham (normoxic air) for five consecutive days followed by follow-up testing at days 8 and 15. In phase two of the study, 10 additional participants underwent the identical IH or placebo administration, and they completed 30 min of daily walking after exposure to each treatment. Results showed that both 1 day and 5 days of IH enhanced walking ability in the form of decreased time to walk 10 m. In addition, IH combined with walking improved walking endurance more than placebo combined with walking. Overall, data demonstrate that a relatively brief exposure to IH combined with task specific training may be the optimal approach to restore function in this population.

Preliminary data from our facility have demonstrated a marked increase in aerobic endurance in response to IH administered immediately prior to completion of electrically stimulated exercise involving partial body weight supported stepping (or standing) (Restorative Therapies RT 600, Baltimore, MD). One woman with complete SCI (age = 28 yr, T2 level of injury, American Spinal Cord Injury Association classification B or incomplete, with sensory but not motor function preserved below the injury level) completed 6 sessions to tolerance over 8 wk and averaged 839.0 ± 64.0 s under stimulation and traveled 480.4 ± 29.1 m. The volunteer then participated in 2 IH + exercise sessions over the next 2 wk, consisting of fifteen 90 s exposures to hypoxia equal to 10% O_2_ separated by normoxic breathing which were completed preceding exercise. Results showed that exercise time under stimulation (1195.0 ± 171.1 s) and distance covered (649.4 ± 96.7 m) were substantially increased. After a 9-day washout period, another exercise session was performed without IH and exercise time under stimulation (917 s) and distance covered (516.6 m) were attenuated. This participant also exhibited nausea and dizziness in response to the initial sessions of exercise which were eliminated after exposure to IH, and these symptoms reoccurred subsequent to washout. Therefore, these unpublished data indicate that IH seems to increase tolerance to electrically stimulated exercise which in the long term may enhance training-induced adaptations.

These few studies clearly show promise of relatively brief exposures to IH interspersed with normoxic breathing in men and women with incomplete SCI on indices of motor and respiratory function without severe side effects. Although not specifically examined, it is likely that maintenance of exposure to IH over longer periods of time may augment health-related outcomes and elicit the potential to reduce mortality and morbidity in this population.

## 5. Potential Mechanisms Explaining Efficacy of Intermittent Hypoxia on Health Status and Physical Function in Persons with Spinal Cord Injury

Human studies documenting a potential benefit of IH on health status in persons with SCI have yet to identify a specific mechanism leading to improved physical function, although BDNF is a likely mediator due to its association with spinal locomotor recovery after SCI [50]. For example, Baker-Herman [36] reported serotonin-dependent increases in BDNF synthesis in ventral spinal segments containing the phrenic nucleus in response to intermittent hypoxia in rats, which could improve respiratory function. Other potential mechanisms which have been identified from animal-based studies to explain effects of intermittent hypoxia on neural function include upregulation of cytoglobin [51], induction of heat shock protein 70 (HSP70) [52], and hypoxia-inducible factor-1-alpha (HIF-1*α*) upregulation [53]. For example, Yuan et al. [54] demonstrated that hypoxic exposure (sixty 30 s cycles of IH equal to 1.5% O_2_ followed by 5 min of normoxia) induced HIF-1*α* accumulation due to increased generation of reactive oxygen species by NADPH oxidase. In addition, increased expression of vascular endothelial growth factor (VEGF) [55] and enhanced growth hormone release [56] have also been shown to be related to neural function in rodents exposed to intermittent hypoxia.

## 6. Directions for Future Research

As previous findings regarding efficacy of IH on physical function in humans with SCI are limited to a small number of short-term trials, much is yet to be understood regarding the utility of this intervention in SCI. Questions remaining to be answered include the following:

1. Can repeated exposure to IH combined with chronic exercise training be a successful weight loss approach in SCI, who are typically overweight or obese?
2. Can a single exposure to IH be used to improve exercise tolerance in various rehabilitation modes including resistance training, electrical stimulation, arm ergometry, and wheelchair ambulation?
3. What is the minimum duration and frequency of IH required to elicit health or function-related benefits?
4. What is the mechanism explaining improved physical function in response to IH in persons with SCI?
5. Can single or repeated exposures to IH elicit potential health-related benefits in persons with complete SCI?
6. Despite no complications reported in existing research in humans with SCI, are there any negative effects of repeated long-term IH exposure?
7. Are there differential effects of IH exposure during rehabilitation of persons with SCI when administered soon after SCI versus in the chronic state of injury?
8. Are there gender differences in responses to short-term IH administration?

## 7. Conclusions

Exposure to relatively brief sessions of intermittent hypoxia has been shown to benefit respiratory, physical, and motor function in humans with incomplete SCI. Combined with the large magnitude of improvements reported in previous research, the duration of these exposures is relatively brief, making IH a robust and time-efficient rehabilitative approach in this population. Although literature in humans with SCI is relatively sparse, it seems that exposure to short-term intermittent hypoxia leads to a variety of physiological benefits with minimal risk, which is contrary to the more popular viewpoint that intermittent hypoxia is a rather high-risk stimulus. Further study is merited to more fully explore the potential of this approach to improve health status in humans with SCI.

## Figures and Tables

**Table 1:** Summary of studies examining effects of acute intermittent hypoxia exposure in humans with spinal cord injury.

Study	Subjects	SCI	IH duration	%O_2_	Parameter measured	Results
Tester et al. (2014) [48]	8 m/w, age = 53 ± 11 yr	C3–T4, chronic	10 days	8.0	Respiratory function	↑ in ventilation with 1 d of AIH

Jaiswal et al. (2014) [42]	1 female, age = 55 yr	C4, chronic	10 days	8.0	Respiratory function	↑ pulmonary function and ventilatory load compensation

Hayes et al. (2014) [44]	19 m/w, age = 43 ± 4 yr	C2–T12, chronic	1 and 5 days	9.0	Walking performance	↑ 6 and 10 min walk time with 1 and 5 d of AIH, especially when combined with overground walking

Trumbower et al. (2012) [43]	13 m/w, age = 46 ± 11 yr	C5–T7, chronic	1 day	9.0	Ankle torque and electromyography	↑ ankle flexion torque and somatic motor output

yr: years old; m: men; w: women; C: cervical; T: thoracic; d: days; IH: intermittent hypoxia; %O_2_: oxygen content of hypoxic inspirate.
